# Pharyngeal reconstruction after total laryngectomy with sliding epiglottis: technical aspects with retrospective review

**DOI:** 10.3389/fonc.2023.1284266

**Published:** 2024-01-08

**Authors:** Aleš Grošelj, Ivana Tancer, Anže Jerman, Jošt Paučič, Luka Pušnik

**Affiliations:** ^1^ Department of Otorhinolaryngology and Cervicofacial Surgery, University Medical Center Ljubljana, Ljubljana, Slovenia; ^2^ Department of Otorhinolaryngology, Faculty of Medicine, University of Ljubljana, Ljubljana, Slovenia; ^3^ Fotona d.o.o., Ljubljana, Slovenia; ^4^ Department of Maxillofacial and Oral Surgery, University Medical Center Ljubljana, Ljubljana, Slovenia; ^5^ Institute of Anatomy, Faculty of Medicine, University of Ljubljana, Ljubljana, Slovenia

**Keywords:** neopharynx, defect reconstruction, primary closure, epiglottoplasty, total laryngectomy

## Abstract

**Introduction:**

Several techniques have been employed for defect reconstruction after total laryngectomy, among others sliding epiglottoplasty. As there is a paucity of data on sliding epiglottoplasty after total laryngectomy, this study aimed to present this reconstruction technique in detail with the retrospective analysis of the patients.

**Methods:**

We retrospectively reviewed single-center medical records of patients who underwent pharyngeal reconstruction after total laryngectomy between 2007-2013, with a follow-up to 2020. The study included patients who had total laryngectomy performed followed by a primary closure or sliding epiglottoplasty. The patients were divided according to the pharyngeal reconstruction technique: sliding epiglottis (*n* = 38) and primary closure (*n* = 120).

**Results:**

The baseline characteristics of patients, TNM stages, and previous treatment strategies did not differ significantly between the sliding epiglottis and primary closure group. The postoperative complication rates, including the pharyngocutaneous fistulae formation and strictures were comparable between the analyzed groups; however, a slightly higher incidence of pharyngocutaneus fistulae was noted within the patients after sliding epiglottoplasty. Overall 3-year survival of patients who underwent the epiglottoplasty and primary closure group were 73.7% *vs*. 57.5%, respectively.

**Conclusions:**

Sliding epiglottoplasty is considered a safe reconstruction technique. Although slightly better outcomes were noted within the epiglottoplasty group, it is still considered inferior to the primary closure. This technique ought to be considered in meticulously selected patients in whom primary closure is not feasible, epiglottis with nearby structures is spared from disease, and when the distal flaps are less appropriate or contraindicated.

## 1 Introduction

Laryngeal cancer remains one of the most common neoplasms in the head and neck region, and surgery remains the mainstay of treatment in locally advanced disease. Total laryngectomy with or without partial pharyngectomy is a procedure carried out as a primary treatment option for advanced laryngeal or hypopharyngeal cancer or as a salvage treatment after recurrence following organ-sparing non-surgical treatment (1–3).

The primary goal of the total laryngectomy is oncologic control of the disease. Along with the locoregional control, the postoperative function improvement of the neopharynx, such as restoration of proper swallowing and adequate speech rehabilitation, is imperative for improving the quality of life (4, 5). In addition, the avoidance of postoperative complications such as infections, pharyngocutaneous fistulae formation, strictures, or aspirations is also of major importance (6). With the advent of local and distal (free or pedicled) flaps that fill the defect of the pharyngeal wall, the armamentarium of reconstruction techniques has improved tremendously and is continuously evolving (4, 7, 8). The flap must be able to withstand the adjuvant radiotherapy and simultaneously be compliant enough to be effectively employed for the restoration of three-dimensional defect (9). As many approaches can be used for defect reconstruction, the understanding of each approach’s advantages and disadvantages is indispensable for providing optimal outcomes.

The sliding epiglottoplasty has been described as an effective local flap technique that enables simple neopharyngeal reconstruction; nonetheless, it frequently goes unnoticed (10–12). In our previously published research (10), a sliding epiglottoplasty has been employed on patients after total laryngectomy; however, the studied group of patients was scarce with a brief follow-up time. This study sought to present the sliding epiglottoplasty procedure in detail with the retrospective analysis of patients who underwent this reconstruction technique while comparing them to the patients in whom the primary closure was used for neopharyngeal formation.

## 2 Materials and methods

### 2.1 Ethical approval

The institutional review board approved this retrospective review of the patient’s medical records. Informed consent was obtained from patients for the acquisition of photographs taken during the operative procedure. Patient confidentiality was maintained in accordance with national standards to protect sensitive patient health information. The study was conducted per the Helsinki Declaration.

### 2.2 Study design

This study retrospectively reviewed patients who underwent total laryngectomy with neopharynx formation (with or without partial pharyngectomy) at the Department of Otorhinolaryngology and Cervicofacial Surgery of the University Medical Centre Ljubljana between March 2007 and July 2013. The patients were divided according to the pharyngeal reconstruction technique that followed total laryngectomy. The epiglottoplasty group consisted of patients with laryngeal or hypopharyngeal squamous cell carcinoma in whom the pharyngeal wall was reconstructed using the sliding epiglottoplasty method as described below. The primary closure group included patients with laryngeal or hypopharyngeal squamous cell carcinoma in whom the neopharynx was formed by primary closure.

The demographic and clinical data including the initial diagnosis, comorbidities, tumor localization and subsites, TNM staging, preoperative treatment strategies, adjuvant treatment, postoperative complications (such as the formation of pharyngocutaneus fistulae and postoperative strictures), voice rehabilitation, and outcomes with a follow-up to July 2020, were retrieved and analyzed. The success of speech rehabilitation was assessed by a speech therapist. Successful speech rehabilitation was defined as the ability of the patient after total laryngectomy to communicate and to phonate as a part of his daily activities. The patients who had another type of carcinoma invading into the larynx, or other distal flaps performed were not included in this study. The patients who were lost during follow-up were excluded from this study.

### 2.3 Epiglottoplasty technique and post-surgical follow-up

The primary closure was considered as a primary reconstruction technique for all patients where this was feasible. The decision regarding the epiglottoplasty technique versus the primary closure was based on the operator’s proficiency in performing the epiglottoplasty procedure and primarily on the presence of a sufficient amount of pharyngeal tissue after tumor removal that allowed the epiglottoplasty procedure. The epiglottoplasty has been derived from Kambic-Sedlacek-Tucker (K-S-T) technique, where epiglottis was used for defect closure after partial laryngectomy (13). When performing this technique, epiglottis ought to be intact with the carcinoma not approaching supraglottic structures (epiglottis, preepiglotic space, superior parts of aryepiglottic folds, or ventricular bands) to provide enough spared tissue that is required for the neopharyngeal reconstruction, whilst retaining a safe resection margin. Thus, patients with supraglottic involvement are not appropriate candidates for this procedure. The pharyngoepiglottic folds, or at least one of them, need to be spared from the disease. In addition, a 1.5 cm wide vertical strip of pharyngeal mucosa must remain intact after tumor removal for the formation of the neopharynx. This flap is sufficient for covering the defects which extend as far as to the esophageal entrance. Since there is no flap rotation, there is a negligible risk of straining the blood vessels.

**Figure 1:**
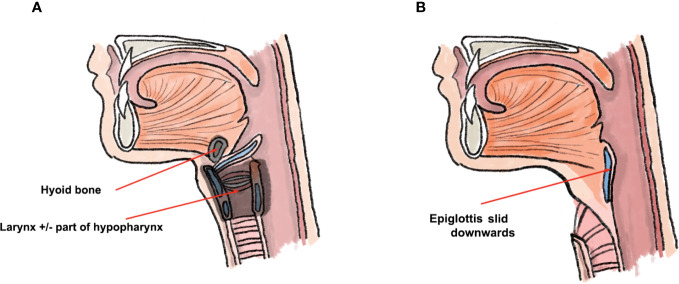
Schematic presentation of epiglottoplasty from the lateral perspective. Figure **(A)** depicts structures which are removed in total laryngectomy, whilst the epiglottis remains connected to the tongue base. In figure **(B)**, the epiglottis is slid down into the pharyngeal defect and neopharynx is formed.

**Figure 2:**
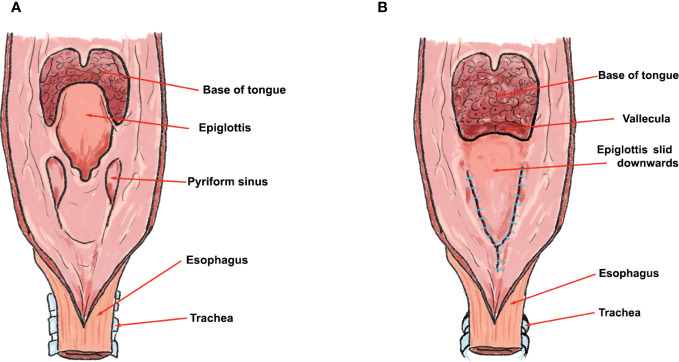
Schematic presentation of epiglottoplasty from the posterior perspective. Figure **(A)** depicts the perspective from posterior pharyngeal wall before the epiglottoplasty, and figure **(B)** the epiglottis being slid and sutured into the pharyngeal defect after total laryngectomy. Note that the closure is made in Y shape with interrupted braided sutures.

**Figure 3:**
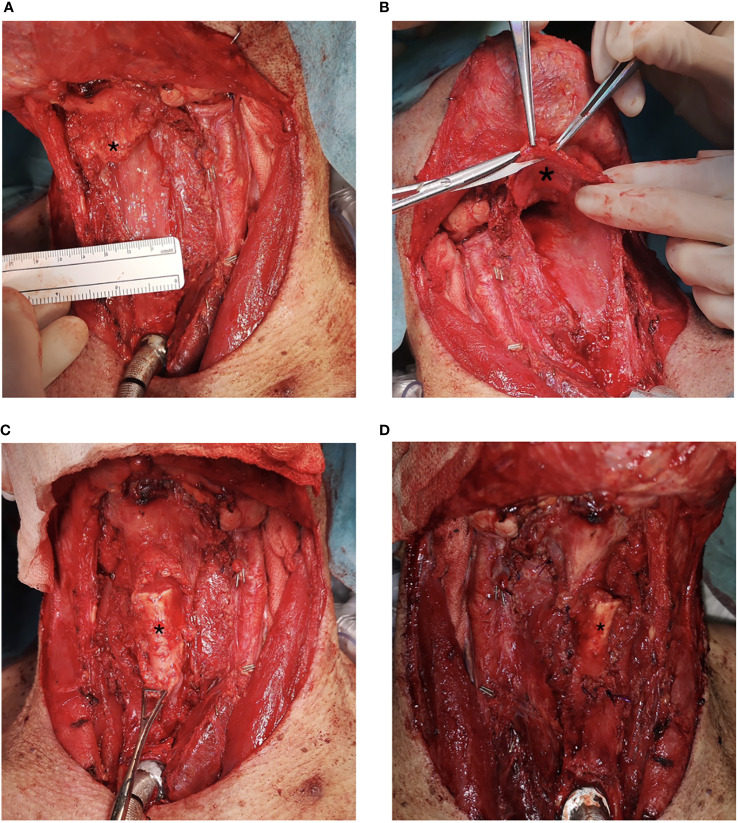
Sliding epiglottis reconstruction. Figure **(A)** depicts a defect after total laryngectomy and partial pharyngectomy with preserved epiglottis (marked with an asterisk *). In figure **(B)**, mobilization of the epiglottis is made. Figure **(C)** displays an epiglottis (marked with an asterisk *) sliding down, filling the pharyngeal defect before formation of neopharynx. Figure **(D)** depicts the formation of the neopharynx with sliding epiglottis.

The sliding epiglottis reconstruction described in brief: after the dissection of an apron flap, strap muscles are divided in order to remove the hyoid bone with preepiglottic space. After exposing the pharyngeal surface of the epiglottic, the dissection of the inferior border near the petioles is made. The resection continues through the aryepiglottic folds. At that point, the tumor can be visualized and dissected, together with the remaining larynx and with/without the pharyngeal structures (depending on the tumor location). The frozen sections are made during the procedure to assess clear operative margins. The procedure of tumor removal exceeds the scope of this article. Following the tumor resection, the pharyngeal surface of the suprahyoid part of the epiglottic cartilage is separated from the overlying mucosa, leaving the epiglottis attached solely to pharyngeal folds and vallecular mucosa. A muco-chondral flap is further displaced downwards in a sliding manner without rotation of the epiglottis to fill the gap (**Figures 1**–**3**). The laryngeal part of mucosa attached to the epiglottis and pharyngoepiglottic folds need to remain uninjured in order to provide sufficient blood supply to the flap. In selected cases, the sliding epiglottis flap can be enlarged with the inclusion of one or both aryepiglottic folds and/or ventricular bands to broaden the epiglottis flap; however, an oncologic safety should be the primary concern. The closure and formation of the neopharynx are made in V or Y shape with interrupted braided sutures (Vicryl Suture 3-0, Ethicon Inc., Johnson & Johnson) and not stapling.

First three years after the procedure, the patients were followed up on bimonthly physical examinations at our clinics. Within the first six months after the procedure, the patients had the ultrasonography of abdominal organs, chest X-ray, and ultrasonography of the neck region performed. In the event of negative radiological findings, these modalities were reperformed at least once per year. From the third to fifth year after the surgery, the patients had regular physical check-ups less frequently, generally once per three to six months, and after the fifth year only once per year. In the event of positive findings at any point, additional diagnostics have been conducted, including computed tomography of the head and neck.

### 2.4 Statistical analysis

Data are given as means ± standard deviations (SD) or proportions when appropriate. The Shapiro-Wilk test was used to evaluate the groups for normality. Because normality and equal variance assumptions were met, numerical data were compared using an independent *t*-test. For enumerable data comparison, a chi-square test was employed. To determine overall survival and disease-specific survival, the date from the operative procedure to the date of the last follow-up or death was used. The 3-year locoregional control rate was calculated in regard to the date of surgery and whether disease relapse was detected during this period. A patient follow-up time was calculated from the procedure until the death or last follow-up (until July 2020). Statistical analysis was performed using GraphPad Prism 10 (GraphPad Software; LLC, San Diego, CA). Differences were deemed statistically significant at *p* < 0.05.

## 3 Results

A total of 158 patients who underwent the total laryngectomy with pharyngeal reconstruction were included in this study. The mean follow-up time was 69.1 ± 50.5 months, ranging from 2 to 162 months. In 38 (24%), the defect was reconstructed using epiglottoplasty, while 120 (76%) patients had the neopharynx formed with a primary closure. The mean age of patients was 61.5 ± 9.3 years with a strong preponderance of male patients (93%). As displayed in **Table 1**, the baseline characteristics of patients did not differ significantly according to the performed reconstruction technique.

**Table 1:** Baseline characteristics of the patients: sliding epiglottis *vs*. primary closure.

		Sliding epiglottis	Primary closure	*p*
Gender	Male	37 (97.4%)	110 (91.7%)	ns
	Female	1 (2.6%)	10 (8.3%)	
Age in years ( $\bar{\text{x}}$ ± SD)		61.6 ± 8.3	61.4 ± 9.6	ns
Arterial hypertension		18 (47.3%)	53 (44.1%)	ns
Presence of diabetes mellitus		3 (7.9%)	9 (7.5%)	ns
Presence of congestive hepatopathy		1 (2.6%)	3 (2.5%)	ns
Presence of vasculitis or autoimmune disease		1 (2.6%)	0 (0.0%)	ns
Heavy alcohol consumption^1^		20 (52.6%)	64 (53.3%)	ns
Active smoking		32 (84.2%)	96 (80.0%)	ns

Data are presented as absolute numbers and proportions and compared using a chi-square or independent t-test test whereas p < 0.05 indicates statistical significance. ^1^Consuming four or more drinks on any day or eight or more per week for women and five or more drinks on any day or fifteen or more per week for men. ns, non-significant.

In the group of patients who had the epiglottoplasty done, hypopharyngeal and laryngeal squamous cell carcinoma were diagnosed in 19 (50%) and 19 patients (50%). In the primary closure group, laryngeal carcinoma was the most common indication for total laryngectomy (*n* = 84; 70%) followed by hypopharyngeal carcinoma (*n* = 36; 30%). There were no statistically significant differences in T, N, or M stages between the groups. No patient was affected by a metastatic disease. The precise distribution of TNM staging is presented in **Tables 2**, **3**, while the subsites of laryngeal and hypopharyngeal tumors are shown in **Table 4**. The proportion of patients with subglottic and pyriform sinus involvement was similar between the groups, while supraglottic involvement was more frequently noted in patients with laryngeal carcinoma of the primary closure group.

**Table 2:** TNM pathological staging: sliding epiglottis *vs*. primary closure.

	Sliding epiglottis^2^ (*n* = 38)			Primary closure^2^ (*n* = 120)			*p*
TNM^1^	Larynx (*n* = 19)	Hypopharynx (*n* = 19)	Σ (*n* = 38)	Larynx (*n* = 84)	Hypopharynx (*n* = 36)	Σ (*n* = 120)	
T1	2 (10.5%)	0 (0.0%)	2 (5.3%)	6 (7.1%)	0 (0.0%)	6 (5.0%)	ns
T2	5 (26.3%)	3 (15.8%)	8 (21.1%)	7 (8.3%)	4 (11.1%)	11 (9.2%)	
T3	5 (26.3%)	13 (68.4%)	18 (47.4%)	38 (45.2%)	20 (55.6%)	58 (48.3%)	
T4	7 (36.8%)	3 (15.8%)	10 (26.3%)	33 (39.3%)	12 (33.3%)	45 (37.5%)	
N0	13 (68.4%)	2 (10.5%)	15 (39.5%)	50 (59.6%)	14 (38.9%)	64 (53.3%)	ns
N1	2 (10.5%)	3 (15.8%)	5 (13.2%)	15 (17.9%)	5 (13.9%)	20 (16.7%)	
N2	3 (15.8%)	14 (73.7%)	17 (44.7%)	19 (22.6%)	16 (44.4%)	35 (29.1%)	
N3	1 (5.3%)	0 (0%)	1 (2.6%)	0 (0.0%)	1 (2.8%)	1 (0.9%)	

Data are presented as absolute numbers and proportions and compared using a chi-square test whereas p < 0.05 indicates statistical significance. ^1^TNM (tumor, node, metastasis) pathological staging according to 8th edition of the American Joint Committee on Cancer. ^2^None of the patients was affected by a metastatic disease. ns, non-significant.

**Table 3:** UICC stage of patients: sliding epiglottis *vs*. primary closure.

		Sliding epiglottis (*n* = 38)			Primary closure (*n* = 120)		
		Larynx (*n* = 19)	Hypopharynx (*n* = 19)	Σ (*n* = 38)	Larynx (*n* = 84)	Hypopharynx (*n* = 36)	Σ (*n* = 120)
TNM^1^ UICC stage	I	2 (10.5%)	0 (0%)	2 (5.3%)	6 (7.1%)	0 (0%)	6 (5.0%)
	II	2 (10.5%)	0 (0%)	2 (5.3%)	6 (7.1%)	3 (8.3%)	9 (7.5%)
	III	5 (26.3%)	4 (21.1%)	9 (23.7%)	31 (36.9%)	8 (22.2%)	39 (32.5%)
	IV_A-C_	10 (52.6%)	15 (78.9%)	25 (65.8%)	41 (48.8%)	25 (69.4%)	65 (54.2%)

^1^TNM (tumor, node, metastasis) pathological staging according to 8th edition of the American Joint Committee on Cancer.

**Table 4:** Subsites of laryngeal and hypopharyngeal tumors: sliding epiglottis *vs*. primary closure.

	Sliding epiglottis		Primary closure	
	Larynx (*n* = 19)	Hypopharynx (*n* = 19)	Larynx (*n* = 84)	Hypopharynx (*n* = 36)
Supraglotic involvement	11 (57.9%)	3 (15.8%)	69 (82.1%)	21 (58.3%)
Glotic involvement	17 (89.5%)	2 (10.5%)	4 (4.7%)	1 (2.8%)
Subglotic involvement	12 (63.1%)	2 (10.5%)	48 (57.1%)	1 (2.8%)
Pyriform sinus involvement	2 (10.5%)	19 (100.0%)	5 (6.0%)	30 (83.3%)
Retrocricoid involvement	2 (10.5%)	9 (47.3%)	2 (2.4%)	18 (50.0%)
Posterior esophageal wall involvement	0 (0.0%)	1 (5.3%)	2 (2.4%)	5 (13.9%)

Data are presented as absolute numbers and proportions, separately for the sliding epiglottis and primary closure group.

The definitive radiation or chemoradiation with subsequent failure was noted in 26% of the epiglottoplasty group, while this proportion was slightly higher (35%) in the primary closure group. There were no significant differences noted when evaluating the preoperative strategies or adjuvant treatment (**Table 5**).

**Table 5:** Treatment strategies: sliding epiglottis *vs*. primary closure.

	Sliding epiglottis (*n* = 38)	Primary closure (*n* = 120)	*p*
Definitive radiotherapy with subsequent failure	6 (15.8%)	28 (23.3%)	ns
Definitive radio-chemotherapy with subsequent failure	4 (10.5%)	14 (11.7%)	ns
Definitive chemotherapy with subsequent failure	0 (0%)	0 (0%)	ns
Previous pharyngeal and/or laryngeal operative procedure	5 (13.2%)	12 (10.0%)	ns
Adjuvant radiotherapy	23 (60.5%)	78 (65.0%)	ns
Adjuvant chemotherapy	6 (15.8%)	21 (17.5%)	ns

Data are presented as absolute numbers and proportions and compared using a chi-square test whereas p < 0.05 indicates statistical significance between sliding epiglottis and primary closure group. ns, non-significant.

A slightly higher incidence of pharyngocutaneus fistulae was noted within the epiglottoplasty group; however, the difference was not statistically significant. The fistulae were noted in 14 patients (37%) of sliding epiglottis and 34 patients (28%) of the primary closure group. The fistulae within the sliding epiglottis group were noted 9.4 ± 4.4 days after the procedure and were present 102.8 ± 141.4 days after the procedure. Within the primary closure group, the fistulae were noted 13.6 ± 9.0 days after the procedure and were present 78.4 ± 88.2 days after the procedure. The postoperative complication rates showed no statistically significant disparities between the groups. In the event of a pharyngocutaneus fistula, a conservative treatment approach has been attempted in all patients according to the institutional practice. This included cessation of oral feeding, insertion of a nasogastric tube or gastrostomy, administration of broad-spectrum antibiotics (amoxicillin with clavulanic acid) and glycopyrrolate or scopolamine to reduce salivation, and removal/curettage of necrotic tissue. None of the patients from the sliding epiglottis group required surgical intervention for fistulae management.

Fifteen patients (39%) from the epiglottoplasty group acquired esophageal speech and ten patients (26%) had the tracheoesophageal prosthesis inserted. In the primary closure group, the proportion of patients who acquired esophageal speech was higher (57%) and the proportion of patients who had the tracheoesophageal prosthesis inserted was lower (8%). Based on the quality assessment of speech therapists, a satisfying speech was achieved in 47% of patients after the epiglottoplasty and 49% of patients after primary closure.

A 3-year locoregional control rate for the epiglottoplasty and primary closure group were 89.5% and 70.8%, respectively. The difference in locoregional control rate between groups was statistically significant (*p* = 0.02). The overall 3-year survival rate for epiglottoplasty and primary closure was 73.7% and 57.5%, respectively (*p* > 0.05). The disease-specific 3-year survival was 80.3% for the epiglottoplasty group and 54.6% for the primary closure group (*p* < 0.05).

## 4 Discussion

Kambic-Sedlacek-Tucker (K-S-T) epiglottoplasty is an established reconstruction technique after laryngectomy (13–15). Hitherto, more authors have implemented this technique after partial or subtotal laryngectomy (16, 17). More recently, Groselj and Fajdiga described this technique as a feasible option for pharyngeal reconstruction after total laryngectomy (10). This study aimed to present the results of sliding epiglottoplasty more in detail with a retrospective analysis of the patients who underwent this procedure. Accordingly, we have analyzed clinical records of patients who underwent total laryngectomy over a six-year-long period. Two groups were proposed and compared based on the performed reconstruction technique: sliding epiglottoplasty versus primary closure.

The results of this study showed that patients who underwent the reconstruction of the neopharynx with the epiglottis flap had better disease-specific survival and better locoregional control rate. This could partly be explained by the fact that epiglottic involvement with cancer bears a higher risk of locoregional recurrences. Similar to hypopharyngeal cancer, such cancers metastasize and recur more often than glottic cancers (18, 19). Conversely, in the group with sliding epiglottoplasty, the proportion of hypopharyngeal involvement was higher than in the group where the neopharynx was formed with primary closure. Concerning this, we can speculate that surgeons performing total laryngectomy with epiglottoplasty might feel more confident in taking wider safety margins, especially in hypopharyngeal tumors, since the remaining epiglottis represent additional tissue for pharyngeal reconstruction.

The association between epiglottoplasty and better outcomes could be partly related to the location and stage of laryngeal/hypopharyngeal cancer, not exclusively the reconstructive procedure per se. Although there were no significant differences in TNM staging between the groups, a minor discrepancy between the T or N stage could also be the driving force behind the difference in oncologic outcomes. The proportion of subglottic and pyriform sinus involvement was similar when comparing the subsite location between the individual groups; however, more tumors with glottic involvement, which has the best oncologic outcomes among laryngeal cancers, was noted in the epiglottoplasty group (20, 21). Despite higher disparities in disease-specific survival, overall survival was more similar in both groups. This could be partially attributed to the synchronous and metachronous second primary cancers and distant metastases which were responsible for the late patients’ death and not analyzed in detail. It should also be acknowledged that the selection of patients appropriate for sliding epiglottis reconstruction has been made biasedly which potentially affected the comparison of herein analyzed groups.

The rates of pyriform sinus involvement, which is recognized as an important risk for pharyngocutaneus fistula formation (22), were similar when comparing the sliding epiglottis and primary closure group. Conversely, the supraglottic tumor invasion which is considered another important risk factor causing pharyngocutaneus fistulae (23, 24) was more frequently observed in primary closure patients. Furthermore, a higher proportion of patients in whom laryngectomy has been performed after definitive radiotherapy or radio-chemotherapy with subsequent failure was noted within the primary closure group. It is imperative to recognize this because the reported incidence of pharyngocutaneus fistulae is higher after the salvage laryngectomy compared to the primary total laryngectomy (22–27). Although we found no statistical significance between the rates of pharyngocutaneus fistulae formation, we believe that more non-biased comparison groups could disclose more evident differences. Hence, a sliding epiglottoplasty should be considered inferior compared to the primary closure regarding the fistulae formation and the employment of this technique should be considered only in selected cases when primary closure is definitely not feasible. In terms of other postoperative complications, the sliding epiglottoplasty and primary closure were comparable as we did not observe any significant disparities between the groups.

The transverse suture lines between the tongue and the remaining pharyngeal wall were avoided when performing the procedure as we hypothesize that such sutures become loose with the tongue movements and thus aid in fistulae formation. Therefore, the broad V form of the suture line was employed to withstand the greater tension. More fistula formations were observed in former cases, afore this study, where closure was performed with monofilament sutures which might cut the cartilage and consequently lead to loosened sutures and fistulae formation. The following led to the modification of this technique with resorbable braided sutures.

Voice rehabilitation has a crucial role in the treatment of patients following total laryngectomy with voice prosthesis becoming a gold standard for voice rehabilitation (4, 28, 29). Although the speech rehabilitation options slightly differed between the epiglottoplasty and primary closure, a satisfying speech was achieved in a similar proportion of both groups. Within our institution, an esophageal speech has been the preferred option for decades which reflects in higher rates; however, nowadays more tracheoesophageal prostheses are inserted.

Herein-described epiglottis flap seems to represent an important technique for pharyngeal reconstruction after total laryngectomy; however, a few conditions have to be met before considering reconstruction with this local flap. As already mentioned, this technique can be appropriate when the primary closure, which is considered the first step of the reconstructive latter, is no longer feasible due to the lack of extra mucosal tissue (30, 31). The pharyngoepiglottic folds have to be spared from the disease and also not resected as the ascending branch of the superior laryngeal artery runs towards the pharyngoepiglottic fold and supplies predominantly the ventral surface of the epiglottis (32).

It is noteworthy that this technique was employed on early-stage patients from both analyzed groups. Such procedures were performed in the past when definitive treatment with radiotherapy failed. Nowadays, in such cases, a conservative approach is imperative and total laryngectomy should be avoided. The implementation of this technique cannot be considered as an efficient replacement for the primary closure; however, sliding epiglottis flap could replace distal flaps in certain cases. For instance, in patients who undergo definitive radiotherapy with subsequent failure, free flaps harvested from outside the irradiated fields are preferred due to the better vascularity in the wound which might aid in healing and decreasing the risk of wound complications (33). One should note that survival of distal flaps depends on adequate blood supply which could be compromised in vascular disease such as peripheral obstructive arterial disease or atherosclerosis (34). In such cases, local flaps are preferred. Although it is known that radiation also affects the cartilage tissue such as the cartilaginous epiglottis (35), it has been shown that survival of the epiglottis flaps after partial laryngectomy is not compromised (10, 36–38). It should be emphasized that local flaps yield less trauma and donor site morbidity while requiring a smaller team of surgeons compared to the distal flaps (10, 39).

We acknowledge that this study had some limitations. First, the selection of patients for epiglottoplasty has been made in a biased manner; however, only to maintain the highest ethical standards and provide the most favorable outcome for each patient. The goal of this study was not to claim the superiority of epiglottoplasty over the primary closure; however, to present cases in which this technique was feasible. Second, there was no comparison with other distal flaps due to the diverse group of patients with distal flaps. Third, a detailed survival analysis has not been performed due to the scarce and partly absent data. Finally, all the patients were gathered from a single center, which might be influenced by local demographics and, more importantly, institutional practice preferences. It would be imperative to overcome the drawbacks of this research with comparative studies.

## 5 Conclusions

The epiglottoplasty, as performed in our institution, is considered a valuable neopharyngeal reconstruction technique. If the epiglottis with nearby structures can be spared during the laryngeal or hypopharyngeal tumor resection and the primary closure is not feasible, we encourage the employment of this easy-to-made local flap.

## Data availability statement

The raw data supporting the conclusions of this article will be made available by the authors, without undue reservation.

## Ethics statement

The studies involving humans were approved by Institutional Review Board at University Clinical Center Ljubljana. The studies were conducted in accordance with the local legislation and institutional requirements. The ethics committee/institutional review board waived the requirement of written informed consent for participation from the participants or the participants’ legal guardians/next of kin because of the retrospective study design with anonymized patient data.

## Author contributions

AG: Conceptualization, Data curation, Funding acquisition, Investigation, Methodology, Resources, Supervision, Visualization, Writing – review & editing. IT: Conceptualization, Data curation, Investigation, Methodology, Writing – original draft. JP: Conceptualization, Validation, Visualization, Writing – review & editing. AJ: Data curation, Investigation, Validation, Visualization, Writing – review & editing. LP: Conceptualization, Data curation, Formal analysis, Investigation, Methodology, Visualization, Writing – original draft, Writing – review & editing.
